# Metabolomic profiles delineate mycolactone signature in Buruli ulcer disease

**DOI:** 10.1038/srep17693

**Published:** 2015-12-04

**Authors:** Fatoumata Niang, Fred S. Sarfo, Michael Frimpong, Laure Guenin-Macé, Mark Wansbrough-Jones, Timothy Stinear, Richard O. Phillips, Caroline Demangel

**Affiliations:** 1 Institut Pasteur, Unité d’Immunobiologie de l’Infection, Paris, France; 2 CNRS URA 1961, Paris, France; 3 Komfo Anokye Teaching Hospital, Kumasi, Ghana; 4 Kumasi Centre for Collaborative Research, Kumasi, Ghana; 5 St George’s University of London, London, United Kingdom; 6 University of Melbourne, Doherty Institute for Infection and Immunity, Melbourne, Australia; 7 Kwame Nkrumah University of Science and Technology, Kumasi, Ghana

## Abstract

Infection of human skin with *Mycobacterium ulcerans*, the causative agent of Buruli ulcer, is associated with the systemic diffusion of a bacterial macrolide named mycolactone. Patients with progressive disease show alterations in their serum proteome, likely reflecting the inhibition of secreted protein production by mycolactone at the cellular level. Here, we used semi-quantitative metabolomics to characterize metabolic perturbations in serum samples of infected individuals, and human cells exposed to mycolactone. Among the 430 metabolites profiled across 20 patients and 20 healthy endemic controls, there were significant differences in the serum levels of hexoses, steroid hormones, acylcarnitines, purine, heme, bile acids, riboflavin and lysolipids. In parallel, analysis of 292 metabolites in human T cells treated or not with mycolactone showed alterations in hexoses, lysolipids and purine catabolites. Together, these data demonstrate that *M. ulcerans* infection causes systemic perturbations in the serum metabolome that can be ascribed to mycolactone. Of particular importance to Buruli ulcer pathogenesis is that changes in blood sugar homeostasis in infected patients are mirrored by alterations in hexose metabolism in mycolactone-exposed cells.

Buruli ulcer (BU) is a necrotizing disease of the skin caused by infection with *Mycobacterium ulcerans*, the third most prevalent mycobacterial pathogen in humans after *M. tuberculosis* and *M. leprae*^1^. How *M. ulcerans* is transmitted to humans is not fully understood, however there is increasing evidence that breaches in the skin barrier and exposure to contaminated environments are both required^2^,^3^,^4^,^5^. Since the 1980s, BU has spread in low-income developing countries of West Africa^6^. If not diagnosed or treated appropriately, it can result in irreversible deformity, functional disability and life-threatening secondary infections. The current diagnosis methods include acid-fast staining, culture or amplification of bacterial DNA from fine needle aspirates, swabs or skin biopsies^7^. Treatment consists of the daily administration of rifampicin and streptomycin for eight weeks^8^,^9^, and excision surgery of large lesions. Although effective, control programs are costly, reactive rather than pro-active, and globally unsuited to field conditions. In order to improve the detection and management of BU, it is essential to improve our understanding of the molecular and cellular mechanisms underpinning BU pathogenesis^10^.

*M. ulcerans* is unique amongst human pathogens in its capacity to produce a polyketide-derived macrolide called mycolactone^11^,^12^,^13^,^14^. Bacterial production of mycolactone is essential for BU formation, as shown by the avirulence of mycolactone-deficient strains of *M. ulcerans* in rodent models of infection. While bacteria grow primarily in host skin tissues, mycolactone gains access to the peripheral circulation^15^,^16^. Foodpad infection of mice with wild-type, but not mycolactone-deficient strains of *M. ulcerans*, induced intrinsic defects in blood T cells evidenced by their incapacity to produce cytokines upon activation *ex vivo*^15^, suggesting that mycolactone modulates the functional biology of T cells at the systemic level. *In vitro*, mycolactone altered the expression of homing receptors by resting T cells, and the production of cytokines by activated T cells, without altering their viability^17^,^18^,^19^. Mycolactone was shown to operate at the post-transcriptional level, and independently of mTOR^17^,^20^. Although its precise mechanism of action remains to be elucidated, there is recent evidence that mycolactone blocks the co-translational translocation of secreted and membrane-bound proteins into the endoplasmic reticulum^21^,^22^. In line with this finding, the proteomic profiling of serum samples of patients with BU showed significant reductions in the level of multiple soluble proteins, including T cell cytokines^23^.

To further explore the physiological consequences of bacterial production of mycolactone in infected hosts, we compared the metabolic perturbations induced by infection with *M. ulcerans* in human hosts to those induced by mycolactone treatment in human cells. Since bacterially-produced mycolactone diffuses from cutaneous lesions into the peripheral circulation, we focused our analysis on serum samples. Jurkat T cells were selected as a model, because leukocytes are exposed to mycolactone during *M. ulcerans* infection^15^,^16^, and Jurkat T cells display the same functional defects as primary T cells upon exposure to mycolactone *in vitro*^17^,^18^,^19^. In addition to provide novel insight into the molecular mechanisms underlying BU pathogenesis, our study delineates mycolactone signature in the serum metabolome of infected hosts.

## Methods

### Ethics statement

The ethics committee at the School of Medical Sciences, Kwame Nkrumah University of Science and Technology, Kumasi, Ghana approved the protocol of this study (CHRPE/11/28/06). All adult subjects provided written informed consent, and a parent or guardian of any child participant provided informed consent on their behalf. The review board also gave approval to document informed consent by use of thumbprints for illiterate participants. Studies using human subjects were performed in accordance with the approved guidelines and regulations.

### Human studies

Two cohorts of patients and age- and gender-matched healthy controls were recruited for the purposes of this study (Table 1). The first cohort was used to compare the metabolic profiles of BU patients and controls from the same community. The second cohort was recruited subsequently, in order to confirm altered cortisol levels in BU patients, and detect an eventual association with lesion severity. Patients were from the middle forest belt of Ashanti Region of Ghana, from Buruli ulcer endemic villages near Tepa Government Hospital (Ahafo Ano North District), Agogo Presbyterian Hospital and Nkawie Government Hospital (Atwima Nwabiagya district). They were included in the study if they met the WHO clinical case definition of *M. ulcerans* disease; were not pregnant; were not receiving antibiotic treatment; had no history of tuberculosis, leprosy, or liver, kidney, or hearing impairment. On the day of clinical diagnosis, fine needle aspirates were taken for PCR amplification of *IS2404* repeat sequence of *M. ulcerans*^24^. Punch biopsy specimens of 4 mm diameter were also stained for acid-fast bacilli and cultured on Lowenstein-Jensen slopes, as previously described^25^. Patients were started on streptomycin 15 mg/kg and rifampicin 10 mg/kg treatment daily for 8 weeks, as recommended by the WHO, at village health posts under direct observation. Blood samples were also collected at the day of clinical diagnosis of BU, before the initiation of antibiotic therapy. Patients were on empty stomach, in an overnight-fasted state. They were asked if they had taken antibiotic or other medication. Only those who had responded negatively and had confirmed BU were subsequently included in the study. Healthy individuals from the same endemic areas also provided serum samples to serve as a comparator. Serum sampling, freezing and storage were performed in a standardized manner, as follows. Blood samples (8 ml) were collected in the field in BD Vacutainer Serum separator tubes, mixed and left to clot according to the manufacturer’s recommendations. Tubes were then transported within 2 h on ice to the laboratory, for centrifugation and serum separation. The recovered serum (3–4 ml) was aliquoted in Eppendorf Safelock^TM^ tubes and stored at −80^o^C. Samples of cohort 1 individuals were shipped to Institut Pasteur (Paris, France) on dry ice, thawed and re-aliquoted in 100 μl-containing Eppendorf Safelock tubes prior to shipping to Metabolon Inc. on dry ice. Serum samples of cohort 2 individuals were shipped to St George’s University of London on dry ice, and assayed for cortisol using Siemens Advia Centaur Competitive Immunoassay and Direct Chemiluminescent Technology.

### Mycolactone

Mycolactone A/B was purified from *M. ulcerans* bacterial cell pellets (strain 1615, ATCC 35840) as previously described^11^. Mycolactone was quantified by measure of absorbance (λ_max_ = 362 nm; log ε = 4.29)^26^, and purity controlled by mass spectrometry. A stock solution (20 μM) was prepared in ethanol solvent that was diluted 1000X for T cell treatments. Controls exposed to the same volume of vehicle were included.

### Cellular studies

Jurkat E6.1 (ATCC TIB-152^TM^) T cells were cultured in RPMI Glutamax^TM^ (Life Technologies), supplemented with 10% heat-inactivated fetal calf serum (FCS) (Invitrogen) and penicillin/streptomycin (100 U*/*ml, 100 μg/ml). Cells in exponential phase of growth were exposed to 20 nM mycolactone (n = 6) or ethanol (n = 5) for 16 h. Cells were recovered and dried by two rounds of centrifugation at 750 g for 3 min, flash-frozen, and stored at −80 °C until analysis.

### Metabolomic profiling

Semi-quantitative metabolomic analyses were performed by Metabolon Inc., as described (http://www.metabolon.com/). On the day of extraction, serum samples (100 μl) or cell pellets (50 μl) were thawed on ice. Proteins were precipitated with methanol, using an automated liquid handler (Hamilton LabStar). The methanol contained four standards, which permitted the monitoring of extraction efficiency. The resulting extract was divided into three fractions that were placed briefly on a TurboVap® (Zymark) to remove the organic solvent, frozen and dried under vacuum. Samples destined to LC/MS analysis were reconstituted in acidic or basic LC-compatible solvents, each of which contained 11 or more injection standards at fixed concentrations. One aliquot was analyzed using acidic positive ion optimized conditions and the other using basic negative ion optimized conditions in two independent injections using separate dedicated columns. Extracts reconstituted in acidic conditions were gradient eluted using water and methanol both containing 0.1% formic acid, while the basic extracts, which also used water/methanol, contained 6.5 mM ammonium bicarbonate. The samples destined for GC/MS analysis were re-dried under vacuum desiccation for a minimum of 24 h prior to being derivatized under dried nitrogen using bistrimethyl-silyl-triflouroacetamide. Technical replicates created from a homogenous pool containing a small amount of all study samples were included. The UPLC-MS/MS platform used a Waters Acquity UPLC with Waters UPLC BEH C18 columns (2.1 × 100 mm, 1.7 μm) and a ThermoFisher LTQ mass spectrometer. GC-MS was performed on a Thermo-Finnigan Trace DSQ fast-scanning single-quadrupole MS. Metabolites were identified by automated comparison of the ion features in the experimental samples to a reference library of chemical standard entries that included retention time, molecular weight (*m*/*z*), preferred adducts, and in-source fragments as well as associated MS spectra. Peaks were quantified by area under the curve measurements. Raw area counts for each metabolite in each sample were normalized to correct for variation resulting from instrument inter-day, tuning differences by the median value for each run-day, therefore setting the medians to 1.0 for each run. Metabolites missing more than one value were excluded from the analysis.

### Statistical analyses

Following log transformation and normalization, Principal Component Analysis (PCA) was used to identify the biochemicals discriminating patients from controls with a false discovery rate (*q*-value) inferior to 0.2. We then used Welch’s two-sample *t*-test to identify biochemicals differing significantly between the two groups (*p* ≤ 0.05). The metabolomic analysis of Jurkat T cells being part of a larger study including multiple treatments, two-way ANOVA with contrasts was used to identify biochemicals differing significantly between mycolactone- and vehicle-treated groups. In both human and cell studies, *q*-values were calculated for each metabolite to take into account multiple comparisons. The GraphPad Prism software (v5.0d, La Jolla, CA) was used for box-and-whisker plot representation, with outliers identified by Tukey’s test.

## Results and Discussion

### Metabolomic profiling of BU

**Figure 1:**
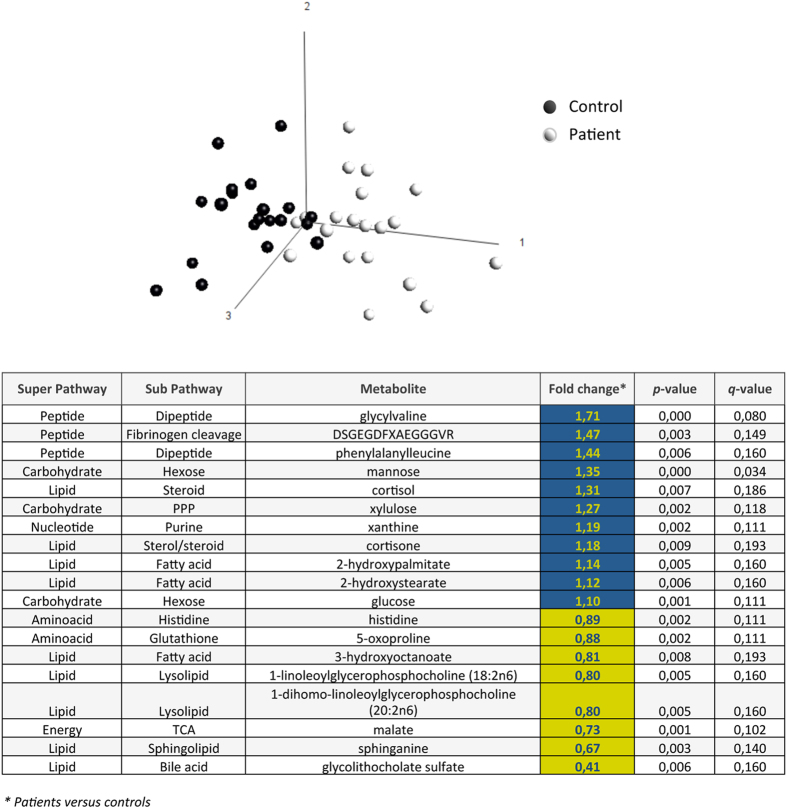
Metabolic signature of BU. PCA scatterplot of serum metabolites in patients with BU and controls. The most discriminating biochemicals (*q-*value ≤ 0.2) are shown, with their *p*-value and variation coefficient (Fold change) across groups (Blue: relatively increased; Yellow: relatively decreased in patients versus controls).

Serum samples were harvested from 20 patients with newly diagnosed BU lesions and 20 age- and gender-matched healthy controls from the same endemic community (Cohort 1, Table 1). Following solvent extraction, samples were split for analysis on liquid or gas chromatography platforms coupled with mass spectrometry. A total of 430 metabolites were identified, whose spectrometric signals were normalized and compared across patients and controls. PCA revealed a separate clustering between the patient and control populations (Fig. 1), showing that BU disease is associated with significant metabolic alterations. Nineteen (4%) metabolites were discriminative (*p*-value < 0.01, *q*-value < 0.2). Among them, 11 were upregulated in patients relative to controls, whereas 8 were downregulated. Intermediates of glycolysis, pentose-phosphate pathway (PPP) and tricarboxylic acid cycle (TCA) were modulated, indicating that energy-generating pathways had been perturbed. Alterations in the peptide, lipid and nucleotide metabolic pathways were also observed. We used a Welch’s two-sample *t*-test (*p* < 0.05) to gain further insight into metabolite differences between groups. Fifty-four metabolites (12%) were present at significantly different levels in patients with BU, compared to controls (Table 2). They clustered into the hexose, fatty acid, lysolipid, steroid hormones, purine and heme metabolism, leading us to examine these pathways in greater detail.

### Hexoses

**Figure 2:**
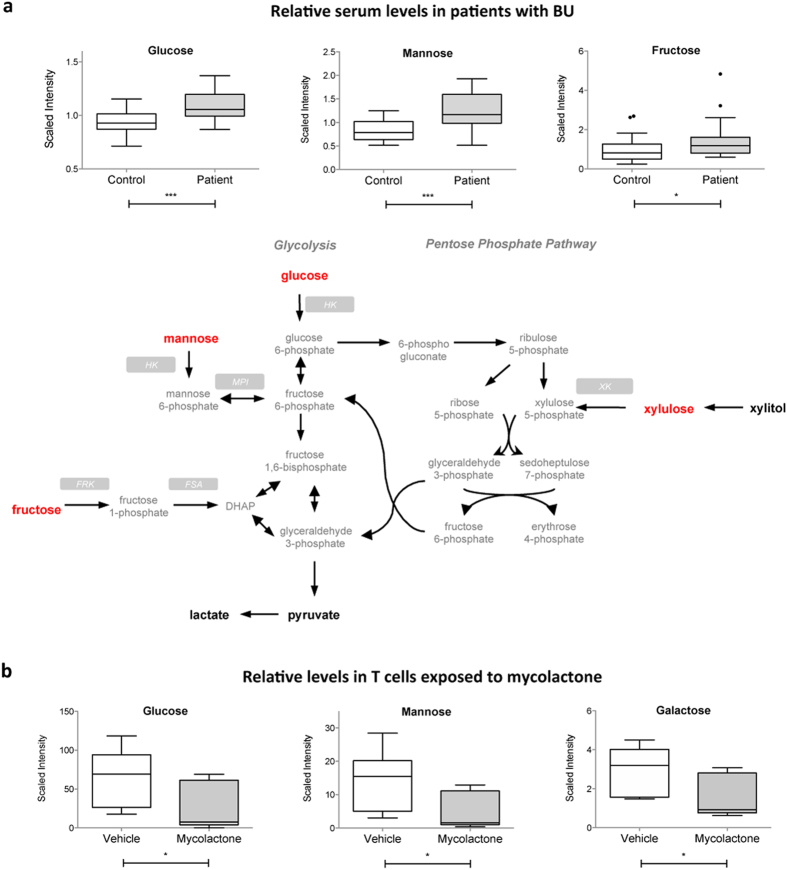
Increased serum hexoses in BU patients mirror decreased hexose concentrations in mycolactone-exposed cells. (**a**) Differential serum levels of the detected hexoses in BU patients versus controls, shown as box and whiskers and in the context of energy-generating metabolic pathways. Biochemicals in bold red were relatively increased in patients versus controls. Those in bold black were detected at comparable levels. Those in grey were not detected. (**b**) Differential concentrations of detected hexoses in mycolactone- and vehicle-treated Jurkat T cells, shown as box and whiskers. *p < 0.05, ***p < 0.001.

Compared to controls, patients with BU displayed elevated levels of all detected hexoses (glucose, fructose and mannose) (Fig. 2a). These sugars enter the cells via common membrane transporters of the solute carrier (SLC)-2 family. The 15–50% increase in serum hexoses may thus indicate defective uptake by SLC2 transporters, or increased hepatic gluconeogenesis. The PPP requires glucose for the generation of pentoses (Fig. 2a). We observed a relative accumulation of xylulose in patient serum (Table 2). Since serum levels of the xylulose precursor xylitol were unchanged, it suggested that generation of PPP intermediate xylulose-5-phosphate might be reduced. Finally, the TCA cycle intermediates citrate and malate were decreased in patients with BU, while alpha-ketoglutarate, succinate and fumarate were unchanged (Supplementary Figure S2 and Table 2). The TCA cycle is essential for the generation of ATP and precursors for various biosynthetic pathways. It requires equilibrated anaplerosis and cataplerosis (for entry and exit of TCA anions, respectively)^27^. In patients with BU, the imbalance between anaplerosis substrates (alpha-ketoglutarate) and cataplerosis substrates (citrate and malate) suggests that TCA cycle function may be impaired.

To determine if some of these effects could result from the action of mycolactone, we profiled the metabolome of Jurkat T cells exposed for 16 h to 20 nM of the purified factor, or vehicle as control. In accordance with previous studies^19^, this treatment decreased the production of membrane receptor CD62L without altering the cell viability (Supplementary Figure S1). Total cell metabolites were extracted and analyzed similar to serum samples, leading to the identification and relative quantification of 292 metabolites. Among them, 59 differed significantly between experimental groups (Table 3). Notably, glucose, galactose and mannose were relatively less concentrated in mycolactone-exposed T cells (Fig. 2b), arguing for a defect in cellular uptake by membrane transporters. Intracellular levels of glucose-1-phosphate and mannose-6-phosphate were downregulated in mycolactone-treated T cells (Table 3), indicative of altered glycolysis. Mycolactone also triggered the intracellular accumulation of acetylcarnitine, propionylcarnitine and butyrylcarnitine (Table 3). On the contrary, serum levels of palmitoylcarnitine and oleoylcarnitine were decreased in patients with BU (Table 2). Because they facilitate the transport of fatty acids across mitochondrial membranes, a rate-limiting step in fatty acid oxidation (FAO), circulating acylcarnitines are clinically-used biomarkers of FAO disorders^28^. At the cellular level, the accumulation of acylcarnitines correlates with reduced oxidation of glucose and insulin resistance^29^. The increased levels of serum hexoses in patients with BU may thus be due, at least partially, to mycolactone-induced defects in hexose uptake and FAO. With the exception of mannose, none of the above-described alterations were observed in patients with TB^30^.

### Steroid hormones

**Figure 3:**
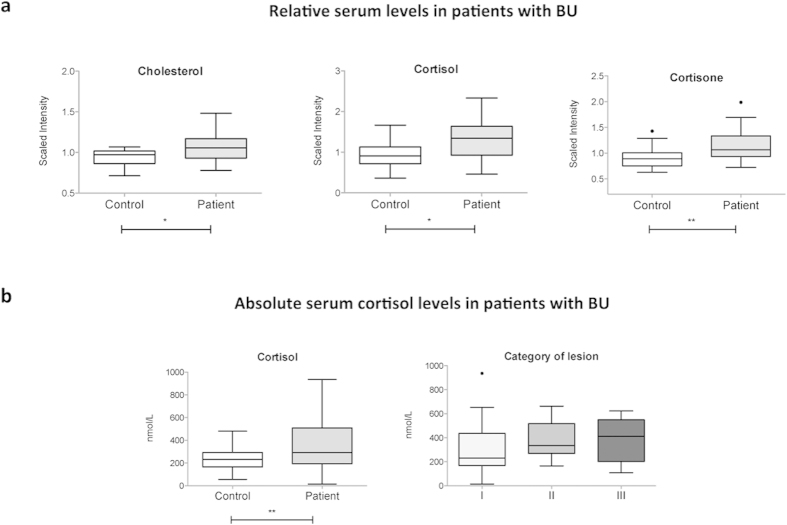
Increased serum glucocorticoids in BU patients. (**a**) Differential serum levels of glucocorticoid hormones and cholesterol in BU patients and controls. (**b**) Absolute concentrations of serum cortisol in BU patients versus controls (left), and patients with different ulcer category (right). Data are presented as box and whiskers. *p < 0.05, **p < 0.01.

Glucocorticoids assist in the regulation of glucose homeostasis through the stimulation of hepatic gluconeogenesis and downregulation of glucose transport systems. There was a relative augmentation in serum cholesterol and downstream glucocorticoid hormones cortisol in patients with BU (Fig. 3a). Cortisone, a conversion product of cortisol with weaker glucocorticoid activity, was also increased whereas other steroidal hormones were not significantly impacted. To validate these findings with an independent and quantitative approach, an additional cohort of patients and controls was assayed for serum cortisol (Cohort 2, Table 1). In agreement with our metabolomics data, the mean cortisol level was higher in patients with BU, compared to controls (Fig. 3b). Although variable, cortisol levels trended higher in patients with more severe lesions (Fig. 3b). No relationship could be demonstrated between serum cortisol and paradoxical reaction, or the clinical form of lesions (nodule, plaque, oedema or ulcer). Together with the data in Fig. 2, these observations suggest that glucocorticoid hormones may be induced in patients with progressive ulcers, in order to raise blood sugars. Since corticosteroids inhibit wound healing, increased circulation in patients may delay their clinical response to antibiotic treatment.

### Purine catabolites

Patients with BU displayed elevated serum levels of the purine catabolites inosine and xanthine (Supplementary Figure S3a and Table 2). These metabolites were not augmented in patients with active TB^30^, suggesting that they do not reflect a general response to mycobacterial infection. Interestingly, opposite variations were observed in T cells exposed to mycolactone (Supplementary Figure S3b and Table 2), whereas adenine and guanine, and the pyrimidine catabolite uracil remained unchanged. Although the underlying molecular mechanism is unclear, increased serum inosine and xanthine may thus constitute specific traits of BU.

### Gamma-glutamyl amino acids

Among the metabolites discriminating patients from controls was the gamma-glutamyl amino acid degradation product 5-oxoproline (Fig. 1), which serum level was relatively lower in patients. Interestingly, patients also displayed reduced levels of gamma-glutamylisoleucine and gamma-glutamylmethionine (Table 2). Gamma-glutamyl amino acids result from the transfer of the gamma-glutamyl moiety of glutathione to acceptor amino acids by the liver enzyme gamma-glutamyl transferase (GGT). We reported previously that BU patients display normal serum GGT^23^. Since isoleucine and methionine were unchanged in patients versus controls in the present study, we can speculate that downregulation of their gamma-glutamyl derivatives is due to limited glutathione availability. Extracellular glutathione results from synthesis, consumption and extrusion by producing cells^31^. Binding of glutamate to cysteine is the first and rate-limiting step in the biosynthesis of this tripeptide. In Jurkat cells, mycolactone had mixed effects on the intracellular levels of gamma-glutamyl amino acids. It did not alter significantly the intracellular levels of cysteine, glutamate nor glutathione (reduced and oxidized forms). Although extracellular glutathione measurements would be required to confirm it, these data suggest that BU-associated alterations in serum gamma-glutamyl amino acids, and potentially glutathione and redox homeostasis, are independent of mycolactone.

### Bile acids

**Figure 4:**
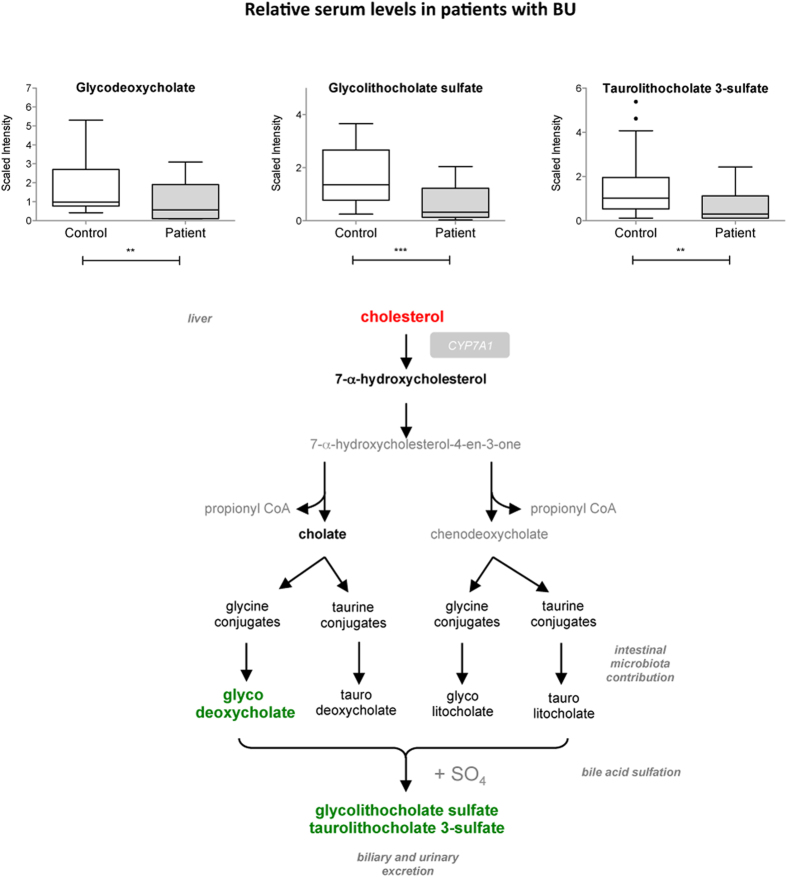
Decreased bile acids levels in the serum of BU patients. Differential serum levels of detected bile acids in patients and controls, shown as box and whiskers and in the context of their metabolic pathways. Biochemicals in bold red were relatively increased in patients versus controls. Those in bold green were relatively decreased. Those in grey were not detected. **p < 0.01, ***p < 0.001.

Bile acids are synthesized from cholesterol by 7-alpha-hydroxylase (CYP7A1) in the liver (Fig. 4). Bile acids facilitate cholesterol elimination, intestinal absorption and excretion of lipids and lipid-soluble molecules. They are also important signaling molecules regulating energy homeostasis, inflammation and liver regeneration^32^. Compared to controls, patients with BU displayed significantly higher levels of cholesterol (Fig. 2a) and normal levels of 7-alpha-hydroxycholesterol and cholate, suggesting that precursors of bile acid synthesis are not limiting. Yet glycodeoxycholate, glycolithocholate sulfate and taurolithocholate 3-sulfate were significantly downregulated in patients with BU (Fig. 4). No variation in bile acids was reported in patients with TB^30^. The reduced levels of bile acids in BU patients may be indicative of decreased synthesis, increased intestinal absorption or urinary excretion. Since bile acid synthesis requires contribution from the microbial community, they may also reflect changes in the intestinal flora.

### Heme products

**Figure 5:**
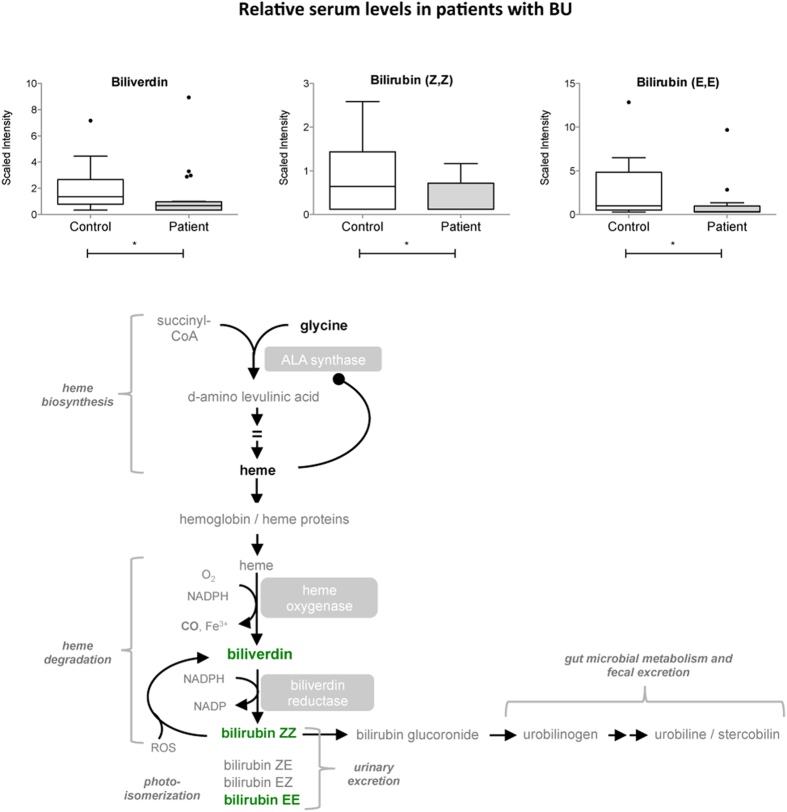
Decreased levels of heme catabolic products in the serum of BU patients. Differential serum levels of biliverdin and bilirubin in patients and controls, shown as box and whiskers and in the context of the heme metabolic pathway. Biochemicals in bold green were relatively decreased in patients versus controls. Those in bold black were detected at comparable levels. Those in grey were not detected. *p < 0.05.

Heme, the most common porphyrin found in the human body, complexes with cellular proteins to form hemoglobin, myoglobin and cytochromes. Heme is synthesized from glycine and succinyl-CoA and can be oxidized into bilirubin and vasodilator carbon dioxide. In patients with BU, heme levels trended higher compared to controls. Conversely, the heme catabolic products biliverdin, bilirubin ZZ and EE were diminished in these subjects (Fig. 5 and Table 2). No such variation was detected in patients with TB, possibly because *M. tuberculosis* possesses its own heme-degrading enzyme MhuD, producing an unusual tetrapyrole called mycobilin^33^. The *M. ulcerans* MhuD gene orthologue (MUL_4167) is a predicted pseudogene due to the introduction of premature stop codon^34^. Consistent with this prediction, no metabolite with a mass corresponding to mycobilin was detected in the serum of patients with BU.

### Riboflavin

Riboflavin (vitamin B2) was recently reported to be upregulated in mosquitoes exposed to live *M. ulcerans*, compared to untreated mosquitoes or mosquitoes exposed to dead bacteria^35^. Interestingly in the present work, patients with BU also displayed increased levels of riboflavin (Table 2). Inspection of the *M. ulcerans* genome predicts an intact riboflavin anabolic pathway. *M. ulcerans* is predicted to possess intact inosine-5′-monophosphate dehydrogenases (e.g. MUL_0901) and GMP synthase (MUL_0913) and the subsequent enzymes to convert these molecules to GTP and enter the riboflavin biosynthesis pathway. Increases in riboflavin are also consistent with the increased levels of the purine metabolism intermediates, inosine and xanthine (see above). The enhanced detection of riboflavin in infected hosts may thus reflect either bacterial growth, or the host response to infection. In any case, the observation that riboflavin levels are associated with *M. ulcerans* infection in both humans and mosquitoes suggest that it could potentially serve as a pathogen-specific correlate of infection.

### Fibrinogen cleavage peptides

Upon vascular injury, soluble fibrinogen is cleaved into insoluble fibrin, which is the main component of blood clots. Fibrinogen A-α cleavage peptides ADSGEGDFXAEGGGVR and DSGEGDFXAEGGGVR were elevated in patients with BU (Supplementary Figure S4 and Table 2), likely reflecting vascular remodeling in lesions. Comparable augmentations were seen in patients with active TB and diabetes^30^,^36^, indicating that this process is not specific to BU.

### Lysolipids

**Figure 6:**
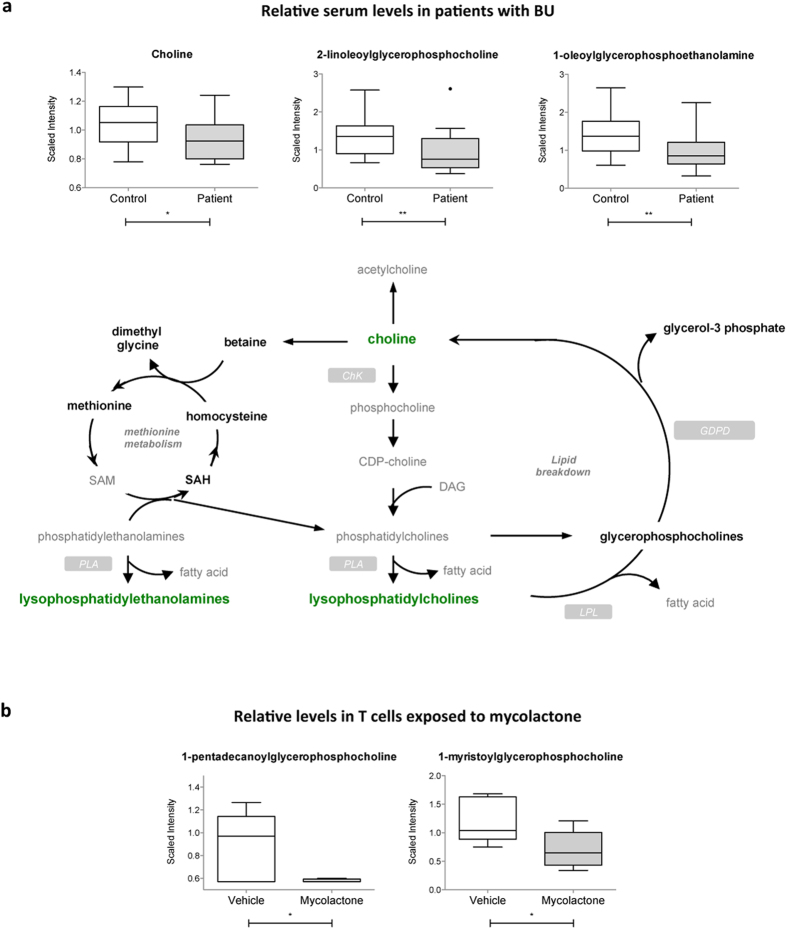
Decreased serum lysolipids in BU patients. (**a**) Differential levels of choline and representative lysolipids in patients and controls, shown as box and whiskers and in the context of their metabolic pathway. Biochemicals in bold green were relatively decreased in patients versus controls. Those in bold black were detected at comparable levels. Those in grey were not detected. (**b**) Differential levels of the detected lysolipids in mycolactone- and vehicle-treated Jurkat T cells, presented as box and whiskers *p < 0.05, **p < 0.01.

Phospholipids (also called glycerophospholipids) are the main lipid constituents of cell membranes. They are a highly diverse family of compounds containing diacylglycerol, a phosphate head group and organic molecules like ethanolamine or choline. Lysolipids and fatty acids are the natural products of their hydrolysis by phospholipases. Compared to controls, patients with BU displayed lower serum levels of choline and all detected lysophosphatidylcholine (LysoPC) compounds (Fig. 6a and Table 2). Lysophosphatidylethanolamines (LysoPE) were comparably impacted. No such variations were reported in patients infected with *M. tuberculosis*^30^, suggesting that they are specific to infection with *M. ulcerans*. In line with this hypothesis, several LysoPC compounds were decreased in mosquitoes exposed to live but not killed preparations of the bacteria^35^. Together with our observations in human patients, these data indicate that *M. ulcerans* interaction with its host may alter phospholipid turnover in biomembranes. In T cells exposed to mycolactone, two LysoPC species were decreased compared to controls (Fig. 6b and Table 3), suggesting that mycolactone may contribute to these changes.

## Conclusion

**Figure 7:**
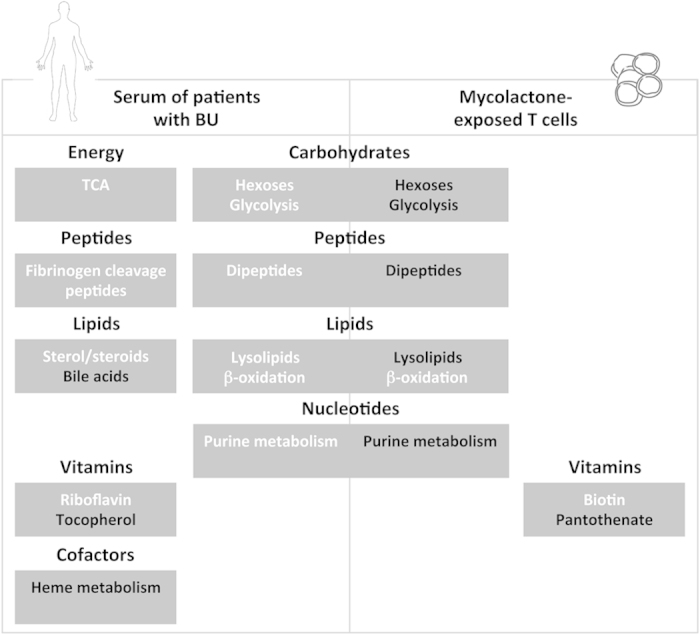
Metabolic alterations in patients with BU and mycolactone-exposed T cells partially overlap. Metabolic alterations are grouped by biochemical pathway/biochemical structure, within boxes entitled with the corresponding metabolic pathway. In white: metabolites in this pathway were detected at higher levels in BU patients *vs* controls, or mycolactone-treated cells *vs* controls. In black: inversely.

Here, we report the metabolomic profiles of serum samples of patients infected with *M. ulcerans,* and mycolactone-exposed cells. Figure 7 summarizes our principal findings, and highlights which metabolites/pathways were modulated in both BU patients and mycolactone-exposed cells. Among them were hexoses, purine products and lysolipids, suggesting that mycolactone released by bacteria interferes with blood cell production of biochemical energy, membrane lipid turnover and degradation of nucleic acids. Interestingly, patients with BU also displayed distinctive downregulation of bile acids and heme products, and upregulation of riboflavin in serum. Intermediates of these metabolic pathways may have potential as biomarkers of BU progression, and inspire new avenues for therapeutic interventions.

## Additional Information

**How to cite this article**: Niang, F. *et al.* Metabolomic profiles delineate mycolactone signature in Buruli ulcer disease. *Sci. Rep.* **5**, 17693; doi: 10.1038/srep17693 (2015).

## Supplementary Material

Supplementary Information

## Figures and Tables

**Table 1:** Human cohort description.

Cohort 1	Healthy controls (n = 20)	Patients with BU (n = 20)
Age, median (range), years	12 (6–35)	13 (7–35)
Sex, no. Male/no. Femelle	12/8	12/8
Ulcer category		
I (lesion size ≤5 cm in widest diameter)		7
II (lesion size ≤15 cm in widest diameter)		13
**Cohort 2**	**Healthy controls (n = 29)**	**Patients with BU (n = 38)**
Age, median (range), years	13 (5–63)	13 (5–75)
Sex, no. Male/no. Femelle	14/15	18/20
Ulcer category		
I (lesion size ≤5 cm in widest diameter)		16
II (lesion size ≤15 cm in widest diameter)		10
III (lesion size >15 cm in widest diameter or multiple lesions)		12

**Table 2:** Metabolic signature of BU in human patients.

Metabolism	Biochemical Pathway	Metabolite	Fold change (Patient *vs* Ctrl)	p-value	q-value
Amino acid	Histidine	histidine	0,85	0,002	0,090
	Lysine	N6-acetyllysine	0,80	0,010	0,172
	Phenylalanine and Tyrosine	p-cresol sulfate	0,64	0,006	0,146
	Cysteine and Methionine	S-methylcysteine	0,67	0,049	0,388
	Glutathione	5-oxoproline	0,84	0,002	0,090
Peptide	Dipeptide	glycylvaline	2,03	0,000	0,023
		isoleucylglycine	1,21	0,038	0,334
		phenylalanylleucine	1,60	0,008	0,169
	Gamma-glutamyl amino acid	gamma-glutamylisoleucine	0,77	0,046	0,386
		gamma-glutamylmethionine	0,81	0,025	0,295
	Fibrinogen cleavage peptide	ADSGEGDFXAEGGGVR	1,47	0,029	0,316
		DSGEGDFXAEGGGVR	1,63	0,002	0,090
Carbohydrate	Hexose	fructose	1,50	0,031	0,316
		mannose	1,51	0,000	0,023
	Glycolysis and Gluconeogenesis	glucose	1,15	0,001	0,090
	Nucleotide Sugar and Pentose	xylulose	1,40	0,003	0,119
Energy	TCA cycle	citrate	0,84	0,012	0,184
		malate	0,67	0,001	0,090
Lipid	Monohydroxy fatty acid	4-hydroxybutyrate (GHB)	0,72	0,048	0,388
		2-hydroxyoctanoate	0,74	0,028	0,316
		3-hydroxyoctanoate	0,74	0,011	0,181
		2-hydroxystearate	1,18	0,011	0,181
		2-hydroxypalmitate	1,21	0,006	0,146
	Beta-oxidation	palmitoylcarnitine	0,66	0,012	0,181
		oleoylcarnitine	0,63	0,008	0,169
	Bile acid	glycodeoxycholate	0,58	0,010	0,172
		glycolithocholate sulfate	0,33	0,000	0,023
		taurolithocholate 3-sulfate	0,42	0,006	0,146
	Glycerolipid	choline	0,91	0,035	0,334
	Lysolipid	1-oleoylglycerophosphoethanolamine	0,71	0,009	0,169
		2-oleoylglycerophosphoethanolamine	0,74	0,032	0,316
		1-linoleoylglycerophosphoethanolamine	0,73	0,028	0,316
		1-palmitoylglycerophosphocholine	0,88	0,042	0,367
		2-palmitoylglycerophosphocholine	0,73	0,024	0,284
		1-oleoylglycerophosphocholine	0,83	0,038	0,334
		1-linoleoylglycerophosphocholine	0,74	0,005	0,146
		2-linoleoylglycerophosphocholine	0,70	0,005	0,146
		1-dihomo-linoleoylglycerophosphocholine	0,73	0,009	0,169
	Monoacylglycerol	1-stearoylglycerol (1-monostearin)	1,25	0,038	0,334
	Sphingolipid	sphinganine	0,55	0,001	0,090
	Sterol/Steroid	cholesterol	1,13	0,020	0,243
		cortisol	1,42	0,018	0,237
		cortisone	1,26	0,007	0,157
Nucleotide	Purine	xanthine	1,27	0,004	0,146
		inosine	2,01	0,004	0,146
Cofactor	Heme	bilirubin (Z,Z)	0,44	0,037	0,334
		bilirubin (E,E)	0,28	0,014	0,204
		biliverdin	0,66	0,019	0,243
Vitamin	Riboflavin	riboflavin (Vitamin B2)	1,82	0,048	0,388
	Tocopherol	gamma-CEHC	0,67	0,017	0,236
Xenobiotic	Chemical	4-methylcatechol sulfate	0,74	0,016	0,221
		hexaethylene glycol	1,20	0,030	0,316
		octaethylene glycol	1,15	0,031	0,316
		pentaethylene glycol	1,13	0,049	0,388

**Table 3:** Metabolic signature of mycolactone in human T cells.

Metabolism	Biochemical Pathway	Metabolite	Fold change (Myco *vs* Ctrl)	p-value	q-value
Amino acid	Glycine, Serine and Threonine	betaine	1,38	0,003	0,028
		beta-alanine	1,41	0,038	0,109
	Glutamate	glutamate	0,77	0,011	0,065
		pyroglutamine	1,31	0,023	0,089
		gamma-aminobutyrate (GABA)	1,43	0,014	0,066
	Histidine	histidine	0,78	0,017	0,072
	Lysine	lysine	0,46	0,008	0,053
		2-aminoadipate	0,53	0,000	0,005
	Phenylalanine and Tyrosine	phenylalanine	0,79	0,026	0,089
		tyrosine	0,71	0,002	0,024
	Tryptophan	tryptophan	0,77	0,025	0,089
	Valine, Leucine and Isoleucine	leucine	0,80	0,031	0,100
		valine	0,81	0,049	0,128
	Cysteine and Methionine	taurine	1,34	0,027	0,089
		methionine	0,76	0,023	0,089
		2-hydroxybutyrate (AHB)	1,25	0,047	0,124
	Urea cycle, Arginine and Proline	dimethylarginine (SDMA + ADMA)	0,65	0,010	0,063
		N-acetylornithine	0,79	0,024	0,089
		argininosuccinate	0,43	0,009	0,056
	Creatine	creatine	1,44	0,001	0,018
	Polyamine	putrescine	1,84	0,001	0,015
Peptide	Dipeptide	glycylproline	0,68	0,036	0,106
		glycylleucine	0,69	0,039	0,109
		glycylthreonine	0,70	0,003	0,031
		prolylglycine	0,58	0,004	0,034
		prolylalanine	0,56	0,004	0,034
		prolylglutamine	0,58	0,005	0,039
		cysteinylglycine	0,58	0,031	0,100
		prolylglutamate	0,62	0,013	0,065
		phenylalanylaspartate	0,61	0,008	0,053
	Gamma-glutamyl amino acid	gamma-glutamylvaline	1,77	0,000	0,004
		gamma-glutamylleucine	1,58	0,001	0,018
		gamma-glutamylisoleucine	1,40	0,012	0,065
		gamma-glutamylmethionine	0,57	0,006	0,047
		gamma-glutamylglutamine	0,62	0,000	0,007
		gamma-glutamylthreonine	2,10	0,000	0,000
Carbohydrate	Aminosugar	Isobar: UDP-acetylglucosamine, UDP-acetylgalactosamine	1,45	0,017	0,072
	Hexose	galactose	0,54	0,012	0,065
		6′-sialyllactose	2,30	0,000	0,005
		mannose	0,39	0,014	0,066
		mannose-6-phosphate	0,53	0,012	0,065
		sorbitol	1,30	0,042	0,114
	Glycolysis	glucose 1-phosphate	0,58	0,033	0,100
		glucose	0,46	0,023	0,089
Lipid	Beta-oxidation	propionylcarnitine	1,80	0,001	0,018
		butyrylcarnitine	2,84	0,002	0,021
	Carnitine	acetylcarnitine	1,34	0,013	0,065
	Glycerolipid	choline phosphate	1,27	0,026	0,089
	Lysolipid	1-myristoylglycerophosphocholine	0,60	0,033	0,100
		1-pentadecanoylglycerophosphocholine	0,64	0,038	0,109
	Neurotransmitter	acetylcholine	9,40	0,000	0,000
	Sterol/Steroid	lathosterol	1,66	0,025	0,089
Nucleotide	Purine	xanthine	0,72	0,002	0,024
		hypoxanthine	0,66	0,020	0,084
		inosine	0,60	0,015	0,070
		inosine 5’-monophosphate (IMP)	0,68	0,042	0,114
		N1-methyladenosine	0,70	0,033	0,100
Cofactor and Vitamin	Biotin	biotin	1,94	0,000	0,000
	Pantothenate and CoA	pantothenate	1,51	0,000	0,004
