# Supplementary material for: Metabolomic profiles delineate mycolactone signature in Buruli ulcer disease
Source: Sci Rep. 2015 Dec 4;5:17693. doi: 10.1038/srep17693 (PMC4669498; doi:10.1038/srep17693)
Supplement: Supplementary Information [file srep17693-s1.pdf]

SUPPLEMENTARY INFORMATION

Metabolomic profiles delineate mycolactone signature in Buruli ulcer disease

Fatoumata Niang, Fred S. Sarfo, Michael Frimpong, Laure Guenin-Macé, Mark Wansbrough-Jones, Timothy Stinear, Richard O. Phillips and Caroline Demangel

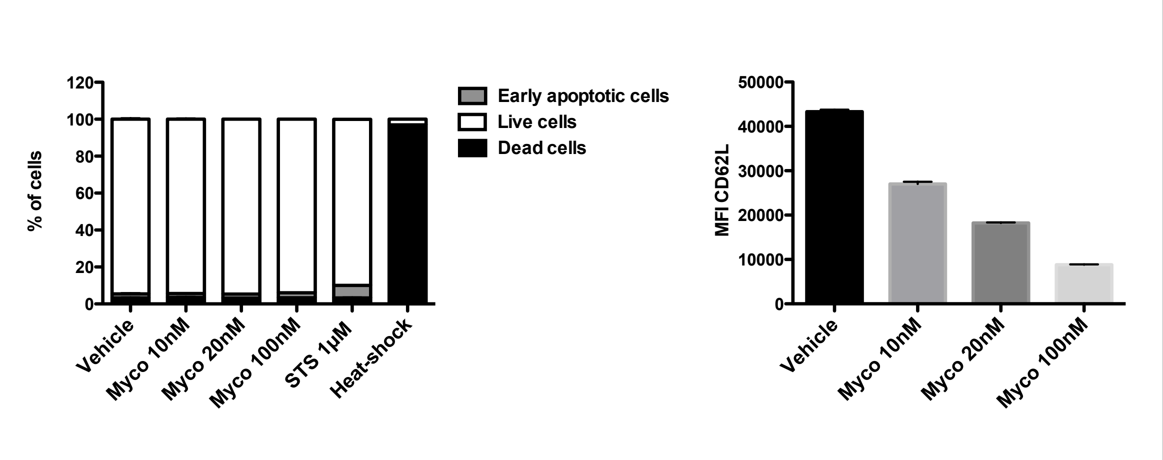

Figure S1: Effects of mycolactone treatment on Jurkat T cells. The viability of Jurkat T cells exposed to mycolactone (Myco) at the indicated concentrations for 16h is shown (Left). Data are percentages of live, early apoptotic and dead cells, as assessed by flow cytometric analysis of Annexin V/propidium iodide staining (Apoptosis Detection Kit, BD Biosciences). Controls include cells treated with mycolactone vehicle, cells treated with the apoptosis inducer staurosporine (STS), and cells submitted to 3 consecutive freeze-thaw cycles (Heat-shock). As a positive control of mycolactone inhibitory activity on protein production, its dose-dependent effect on T cell expression of CD62L was monitored in parallel (Right).

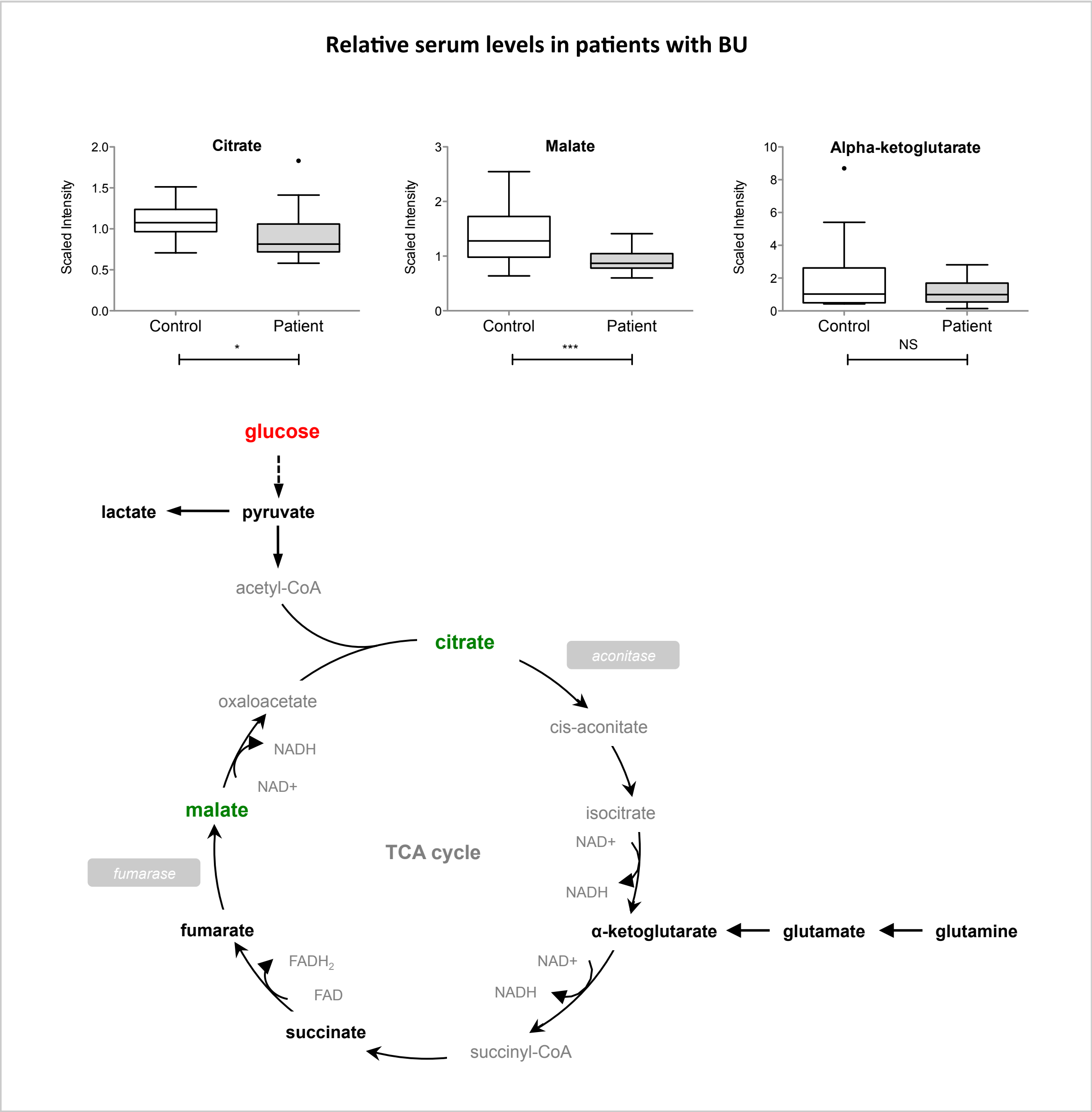

Figure S2: Selective decrease in TCA intermediates in the serum of BU patients. Differential serum levels of TCA intermediates in patients and controls, presented as Box and Whiskers and in the context of their metabolic pathway. Metabolites in bold green were relatively decreased in patients versus controls. Those in bold black were detected at comparable levels. Those in grey were not detected. *p<0.05, ***p<0.001, NS: not significant.

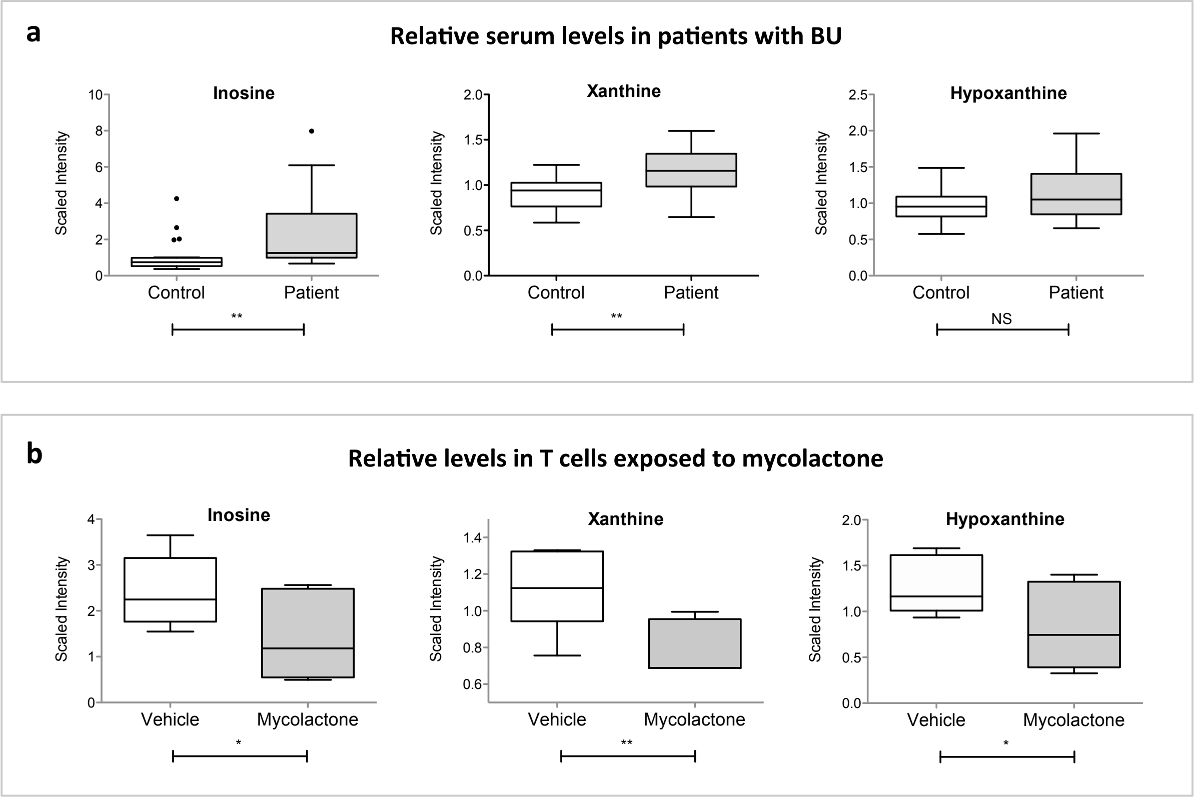

Figure S3: Opposite variation profiles of purine catabolites in the serum of BU patients and mycolactone-treated cells. Differential serum levels of inosine, xanthine and hypoxanthine in patients with BU and controls (**a**), and in mycolactone- and vehicle-treated Jurkat T cells (**b**). Data are presented as Box and Whiskers, *p<0.05, **p<0.01, NS: not significant.

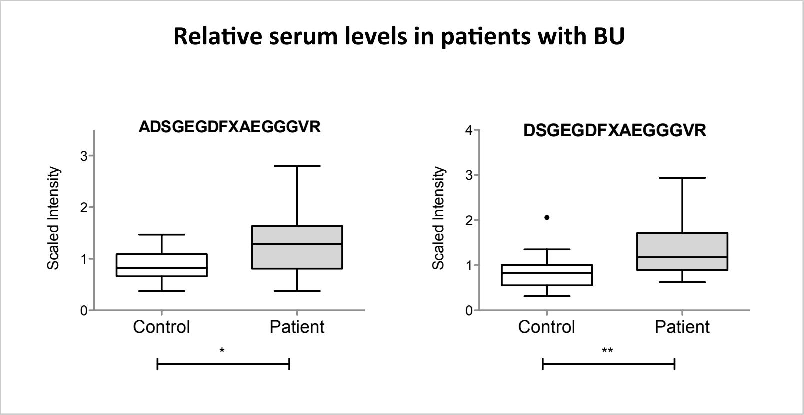

Figure S4: Accumulation of fibrinogen cleavage peptides in the serum of BU patients. Differential serum levels of fibrinogen A-α cleavage peptides in patients and controls, presented as Box and Whiskers. *p<0.05, **p<0.01.
